# 2-Benzyl­sulfanyl-4-[(4-methyl­phen­yl)sulfan­yl]-6-pentyl­pyrimidine-5-carbonitrile

**DOI:** 10.1107/S1600536812025275

**Published:** 2012-06-13

**Authors:** Ali A. El-Emam, Omar A. Al-Deeb, Nasser R. El-Brollosy, Seik Weng Ng, Edward R. T. Tiekink

**Affiliations:** a Department of Pharmaceutical Chemistry, College of Pharmacy, King Saud University, Riyadh 11451, Saudi Arabia; b Department of Chemistry, University of Malaya, 50603 Kuala Lumpur, Malaysia; c Chemistry Department, Faculty of Science, King Abdulaziz University, PO Box 80203 Jeddah, Saudi Arabia

## Abstract

In the title compound, C_24_H_25_N_3_S_2_, the S-bound benzene rings have orthogonal [dihedral angle = 85.31 (9)°] and splayed [67.92 (11)°] orientations with respect to the pyrimidine ring; the dihedral angle between the benzene rings is 48.18 (12)°. The pentyl group has an extended all-*trans* conformation and lies to one side of the pyrimidine ring [the N_py_—C_py_—C_p_—C_p_ torsion angle = −85.7 (2)°; py = pyrimidine and p = pent­yl].

## Related literature

For the chemotherapeutic activity of pyrimidine derivatives see: Ghoshal & Jacob (1997 ▶); De Corte (2005 ▶); Al-Omar *et al.* (2010 ▶); Al-Abdullah *et al.* (2011 ▶); Al-Turkistani *et al.* (2011 ▶). For a related pyrimidine structure, see: El-Emam *et al.* (2012 ▶).
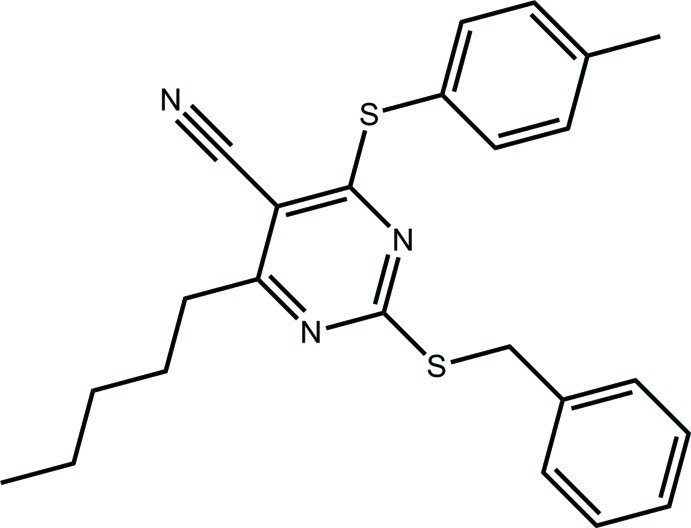


## Experimental

### 

#### Crystal data

C_24_H_25_N_3_S_2_
*M* *_r_* = 419.59
Monoclinic, 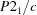

*a* = 9.9178 (2) Å
*b* = 8.2235 (2) Å
*c* = 28.4388 (8) Å
β = 96.115 (2)°
*V* = 2306.24 (10) Å^3^
*Z* = 4
Cu *K*α radiation
μ = 2.19 mm^−1^
*T* = 294 K
0.40 × 0.20 × 0.10 mm

#### Data collection

Agilent SuperNova Dual diffractometer with Atlas detector
Absorption correction: multi-scan (*CrysAlis PRO*; Agilent, 2012 ▶) *T*_min_ = 0.689, *T*_max_ = 1.000
9523 measured reflections
4746 independent reflections
3720 reflections with *I* > 2σ(*I*)
*R*_int_ = 0.017

#### Refinement

*R*[*F* ^2^ > 2σ(*F* ^2^)] = 0.044
*wR*(*F* ^2^) = 0.138
*S* = 1.03
4746 reflections
264 parameters
H-atom parameters constrained
Δρ_max_ = 0.18 e Å^−3^
Δρ_min_ = −0.24 e Å^−3^

### 

Data collection: *CrysAlis PRO* (Agilent, 2012 ▶); cell refinement: *CrysAlis PRO*; data reduction: *CrysAlis PRO*; program(s) used to solve structure: *SHELXS97* (Sheldrick, 2008 ▶); program(s) used to refine structure: *SHELXL97* (Sheldrick, 2008 ▶); molecular graphics: *ORTEP-3 for Windows* (Farrugia, 1997 ▶) and *DIAMOND* (Brandenburg, 2006 ▶); software used to prepare material for publication: *publCIF* (Westrip, 2010 ▶).

## Supplementary Material

Crystal structure: contains datablock(s) global, I. DOI: 10.1107/S1600536812025275/zl2483sup1.cif

Structure factors: contains datablock(s) I. DOI: 10.1107/S1600536812025275/zl2483Isup2.hkl

Supplementary material file. DOI: 10.1107/S1600536812025275/zl2483Isup3.cml

Additional supplementary materials:  crystallographic information; 3D view; checkCIF report

## Figures and Tables

**Fig. 1.**
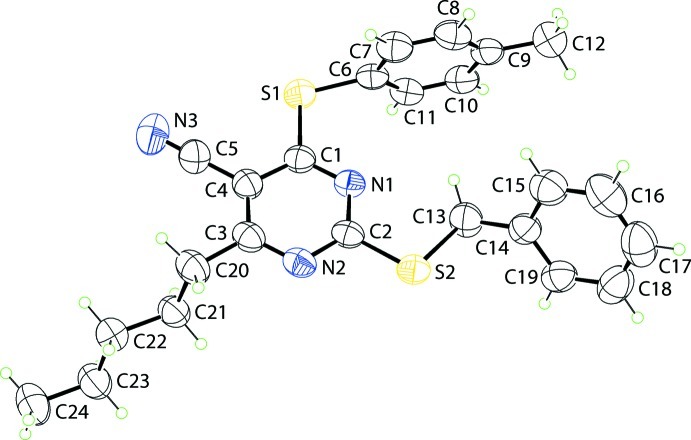
The molecular structure of the title compound showing the atom-labelling scheme and displacement ellipsoids at the 35% probability level.

**Fig. 2.**
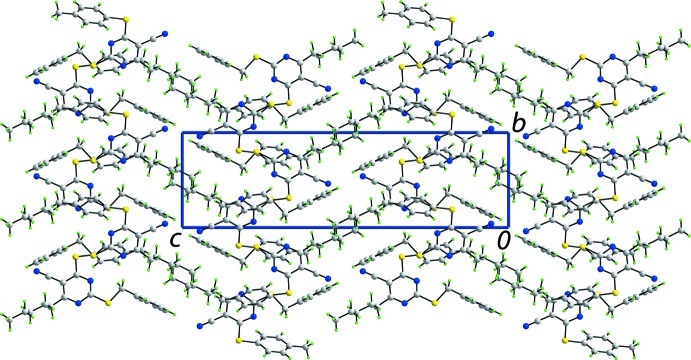
A view in projection down the a axis of the unit-cell contents for the title compound.

## supplementary crystallographic information

### Comment

The ability of of pyrimidine derivatives to inhibit vital enzymes responsible for DNA bio-synthesis is the reason behind their chemotherapeutic efficacy. Thus, several pyrimidine non-nucleoside derivatives exhibit anti-cancer (Ghoshal & Jacob, 1997), anti-viral (De Corte, 2005) and anti-bacterial activities (Al-Abdullah *et al.*, 2011). The synthesis and crystal structure determination of the title compound, was undertaken in connection with on-going studies of the chemical, pharmacological and structural properties of pyrimidine derivatives (Al-Omar *et al.*, 2010; Al-Turkistani *et al.*, 2011; El-Emam *et al.* 2012).

With reference to the pyrimidine ring (r.m.s. deviation = 0.011 Å) in the title compound, Fig. 1, the S1- and S2-bound benzene rings form dihedral angles of 85.31 (9) and 67.92 (11)°, respectively, which indicate an orthogonal and a splayed orientation, respectively; the dihedral angle between the benzene rings is 48.18 (12)°. The pentyl group lies to one side of the pyrimidine ring with the N2—C3—C20—C21 torsion angle being -85.7 (2)°. The remaining chain has an extended all-*trans* conformation [the C20—C21—C22—C23 and C21—C22—C23—C24 torsion angles are -173.8 (2) and 179.6 (2)°, respectively].

No specific intermolecular interactions are evident in the crystal structure. Globally, molecules lie in layers in the *ab* plane which stack along the *c* axis, Fig. 2.

### Experimental

To a solution of 2-(benzylsulfanyl)-4-chloro-6-(*n*-pentyl)pyrimidine-5-carbonitrile (665 mg, 2 mmol) in dry pyridine (3 ml), 4-thiocresol (248 mg, 2 mmol) was added and the mixture was heated under reflux for 6 h. On cooling, the solvent was then distilled off *in vacuo* and water (5 ml) was added to the residue. The separated precipitate was collected by filtration, washed with cold water, dried and recrystallized from ethanol to yield 747 mg (89%) of the title compound as colourless crystals. M.p.: 384–386 K. Single crystals suitable for X-ray analysis were obtained by slow evaporation of a solution of the title compound in CHCl_3_:EtOH (1:1, 5 ml) at room temperature. ^1^H NMR (DMSO-d_6_, 500.13 MHz): δ 0.85 (t, 3H, CH_3_, J = 7.0 Hz), 1.26–1.34 (m, 4H, C*H*_2_C*H*_2_CH_3_), 1.64–1.69 (m, 2H, C*H*_2_CH_2_CH_2_CH_3_), 2.27 (s, 3H, Ar—CH_3_), 2.76 (t, 2H, C*H*_2_CH_2_CH_2_CH_2_CH_3_, J = 7.0 Hz), 4.02 (s, 2H, CH_2_S), 6.98–6.99 (m, 2H, Ar—H), 7.21–7.23 (m, 3H, Ar—H), 7.28 (d, 2H, Ar—H, J = 8.0 Hz), 7.51 (d, 2H, Ar—H, J = 8.0 Hz). ^13^C NMR (DMSO-d_6_, 125.76 MHz): δ 14.16 (CH_3_), 21.29 (*C*H_2_CH_3_), 22.29 (ArCH_3_), 27.39 (*C*H_2_CH_2_CH_3_), 31.13 (*C*H_2_CH_2_CH_2_CH_3_), 34.51 (*C*H_2_CH_2_CH_2_CH_2_CH_3_), 36.12 (CH_2_S), 99.14 (C-5), 114.69 (CN), 122.19, 127.56, 128.74, 129.01, 130.67, 136.20, 137.58, 140.99 (Ar—C), 172.81, 173.15, 173.37 (C-2, C-4 & C-6).

### Refinement

Carbon-bound H-atoms were placed in calculated positions [C—H = 0.93 to 0.97 Å, *U*_iso_(H) = 1.2–1.5*U*_eq_(C)] and were included in the refinement in the riding model approximation.

### Crystal data

**Table:**

C_24_H_25_N_3_S_2_	*F*(000) = 888
*M**_r_* = 419.59	*D*_x_ = 1.208 Mg m^−^^3^
Monoclinic, *P*2_1_/*c*	Cu *K*α radiation, λ = 1.54184 Å
Hall symbol: -P 2ybc	Cell parameters from 3374 reflections
*a* = 9.9178 (2) Å	θ = 4.6–76.5°
*b* = 8.2235 (2) Å	µ = 2.19 mm^−^^1^
*c* = 28.4388 (8) Å	*T* = 294 K
β = 96.115 (2)°	Prism, colourless
*V* = 2306.24 (10) Å^3^	0.40 × 0.20 × 0.10 mm
*Z* = 4	

### Data collection

**Table:**

Agilent SuperNova Dual diffractometer with Atlas detector	4746 independent reflections
Radiation source: SuperNova (Cu) X-ray Source	3720 reflections with *I* > 2σ(*I*)
Mirror monochromator	*R*_int_ = 0.017
Detector resolution: 10.4041 pixels mm^-1^	θ_max_ = 76.7°, θ_min_ = 5.6°
ω scan	*h* = −12→10
Absorption correction: multi-scan (*CrysAlis PRO*; Agilent, 2012)	*k* = −10→10
*T*_min_ = 0.689, *T*_max_ = 1.000	*l* = −35→29
9523 measured reflections	

### Refinement

**Table:**

Refinement on *F*^2^	Secondary atom site location: difference Fourier map
Least-squares matrix: full	Hydrogen site location: inferred from neighbouring sites
*R*[*F*^2^ > 2σ(*F*^2^)] = 0.044	H-atom parameters constrained
*wR*(*F*^2^) = 0.138	*w* = 1/[σ^2^(*F*_o_^2^) + (0.0721*P*)^2^ + 0.1537*P*] where *P* = (*F*_o_^2^ + 2*F*_c_^2^)/3
*S* = 1.03	(Δ/σ)_max_ = 0.001
4746 reflections	Δρ_max_ = 0.18 e Å^−^^3^
264 parameters	Δρ_min_ = −0.24 e Å^−^^3^
0 restraints	Extinction correction: *SHELXL*, Fc^*^=kFc[1+0.001xFc^2^λ^3^/sin(2θ)]^-1/4^
Primary atom site location: structure-invariant direct methods	Extinction coefficient: 0.0040 (4)

### Special details

**Table:**

Geometry. All e.s.d.'s (except the e.s.d. in the dihedral angle between two l.s. planes) are estimated using the full covariance matrix. The cell e.s.d.'s are taken into account individually in the estimation of e.s.d.'s in distances, angles and torsion angles; correlations between e.s.d.'s in cell parameters are only used when they are defined by crystal symmetry. An approximate (isotropic) treatment of cell e.s.d.'s is used for estimating e.s.d.'s involving l.s. planes.
Refinement. Refinement of *F*^2^ against ALL reflections. The weighted *R*-factor *wR* and goodness of fit *S* are based on *F*^2^, conventional *R*-factors *R* are based on *F*, with *F* set to zero for negative *F*^2^. The threshold expression of *F*^2^ > σ(*F*^2^) is used only for calculating *R*-factors(gt) *etc*. and is not relevant to the choice of reflections for refinement. *R*-factors based on *F*^2^ are statistically about twice as large as those based on *F*, and *R*- factors based on ALL data will be even larger.

### Fractional atomic coordinates and isotropic or equivalent isotropic displacement parameters (Å^2^)

**Table:**

	*x*	*y*	*z*	*U*_iso_*/*U*_eq_	
S1	0.78263 (6)	0.31009 (7)	0.67270 (2)	0.0889 (2)	
S2	0.60772 (5)	0.79595 (6)	0.773134 (19)	0.08036 (18)	
N1	0.68705 (13)	0.56138 (17)	0.71826 (5)	0.0665 (3)	
N2	0.58228 (15)	0.79774 (19)	0.68118 (6)	0.0764 (4)	
N3	0.6892 (3)	0.4609 (4)	0.55333 (8)	0.1323 (9)	
C1	0.70156 (16)	0.4995 (2)	0.67609 (7)	0.0696 (4)	
C2	0.62755 (16)	0.7066 (2)	0.71888 (7)	0.0678 (4)	
C3	0.59644 (18)	0.7347 (3)	0.63894 (8)	0.0775 (5)	
C4	0.65498 (17)	0.5814 (3)	0.63450 (7)	0.0745 (5)	
C5	0.6733 (2)	0.5142 (3)	0.58931 (8)	0.0940 (6)	
C6	0.84843 (18)	0.2845 (2)	0.73261 (7)	0.0729 (5)	
C7	0.97186 (19)	0.3532 (3)	0.74887 (8)	0.0858 (6)	
H7	1.0217	0.4096	0.7283	0.103*	
C8	1.0201 (2)	0.3376 (3)	0.79569 (9)	0.0886 (6)	
H8	1.1030	0.3847	0.8064	0.106*	
C9	0.9493 (2)	0.2538 (3)	0.82738 (8)	0.0825 (5)	
C10	0.8272 (2)	0.1824 (3)	0.80980 (8)	0.0846 (6)	
H10	0.7786	0.1229	0.8301	0.102*	
C11	0.77708 (18)	0.1978 (2)	0.76337 (8)	0.0787 (5)	
H11	0.6948	0.1497	0.7525	0.094*	
C12	1.0013 (3)	0.2400 (4)	0.87868 (10)	0.1206 (9)	
H12A	1.0233	0.1287	0.8861	0.181*	
H12B	0.9329	0.2767	0.8977	0.181*	
H12C	1.0811	0.3059	0.8851	0.181*	
C13	0.6781 (3)	0.6417 (3)	0.81484 (8)	0.0932 (6)	
H13A	0.6102	0.5595	0.8189	0.112*	
H13B	0.7550	0.5892	0.8028	0.112*	
C14	0.7221 (2)	0.7210 (2)	0.86107 (7)	0.0781 (5)	
C15	0.8585 (3)	0.7470 (4)	0.87499 (10)	0.1021 (7)	
H15	0.9228	0.7152	0.8553	0.123*	
C16	0.8998 (3)	0.8197 (4)	0.91789 (13)	0.1233 (10)	
H16	0.9918	0.8365	0.9267	0.148*	
C17	0.8086 (4)	0.8667 (4)	0.94725 (11)	0.1249 (10)	
H17	0.8378	0.9148	0.9761	0.150*	
C18	0.6744 (4)	0.8435 (3)	0.93449 (10)	0.1126 (8)	
H18	0.6112	0.8762	0.9545	0.135*	
C19	0.6317 (2)	0.7711 (3)	0.89167 (9)	0.0911 (6)	
H19	0.5394	0.7557	0.8833	0.109*	
C20	0.5482 (2)	0.8344 (3)	0.59621 (8)	0.0928 (6)	
H20A	0.5996	0.8045	0.5704	0.111*	
H20B	0.5653	0.9484	0.6033	0.111*	
C21	0.3988 (2)	0.8112 (3)	0.58083 (8)	0.0843 (5)	
H21A	0.3462	0.8583	0.6043	0.101*	
H21B	0.3785	0.6959	0.5787	0.101*	
C22	0.3585 (2)	0.8912 (3)	0.53285 (7)	0.0885 (6)	
H22A	0.3891	1.0033	0.5342	0.106*	
H22B	0.4049	0.8360	0.5091	0.106*	
C23	0.2100 (2)	0.8882 (4)	0.51799 (9)	0.1024 (7)	
H23A	0.1632	0.9429	0.5417	0.123*	
H23B	0.1792	0.7762	0.5162	0.123*	
C24	0.1729 (3)	0.9695 (4)	0.47047 (8)	0.1191 (9)	
H24A	0.0762	0.9674	0.4629	0.179*	
H24B	0.2151	0.9124	0.4465	0.179*	
H24C	0.2038	1.0802	0.4719	0.179*	

### Atomic displacement parameters (Å^2^)

**Table:**

	*U*^11^	*U*^22^	*U*^33^	*U*^12^	*U*^13^	*U*^23^
S1	0.0998 (4)	0.0797 (3)	0.0874 (4)	0.0206 (3)	0.0105 (3)	−0.0054 (3)
S2	0.0877 (3)	0.0652 (3)	0.0881 (3)	0.0125 (2)	0.0093 (2)	0.0008 (2)
N1	0.0611 (7)	0.0595 (7)	0.0790 (9)	0.0001 (6)	0.0086 (6)	0.0046 (7)
N2	0.0701 (8)	0.0691 (9)	0.0894 (11)	0.0054 (7)	0.0059 (7)	0.0147 (8)
N3	0.160 (2)	0.153 (2)	0.0858 (13)	0.0146 (18)	0.0225 (14)	−0.0026 (15)
C1	0.0595 (8)	0.0677 (9)	0.0822 (11)	−0.0005 (7)	0.0107 (8)	0.0044 (9)
C2	0.0556 (8)	0.0622 (9)	0.0855 (11)	−0.0011 (7)	0.0069 (7)	0.0079 (8)
C3	0.0629 (9)	0.0820 (12)	0.0871 (13)	−0.0012 (9)	0.0061 (8)	0.0159 (10)
C4	0.0656 (9)	0.0816 (11)	0.0763 (11)	−0.0025 (8)	0.0076 (8)	0.0072 (9)
C5	0.0948 (14)	0.1054 (17)	0.0820 (13)	0.0045 (12)	0.0112 (11)	0.0084 (13)
C6	0.0685 (9)	0.0600 (9)	0.0909 (12)	0.0119 (8)	0.0116 (8)	0.0009 (9)
C7	0.0723 (11)	0.0789 (12)	0.1077 (16)	−0.0043 (9)	0.0165 (11)	0.0062 (11)
C8	0.0663 (10)	0.0827 (12)	0.1157 (17)	−0.0002 (9)	0.0046 (10)	−0.0039 (12)
C9	0.0767 (11)	0.0776 (11)	0.0935 (14)	0.0201 (10)	0.0096 (10)	−0.0055 (10)
C10	0.0753 (11)	0.0795 (12)	0.1015 (15)	0.0072 (9)	0.0207 (10)	0.0103 (11)
C11	0.0645 (9)	0.0689 (10)	0.1023 (15)	0.0031 (8)	0.0074 (9)	0.0035 (10)
C12	0.1118 (19)	0.148 (3)	0.0993 (18)	0.0205 (18)	−0.0022 (14)	−0.0077 (18)
C13	0.1218 (17)	0.0681 (11)	0.0882 (14)	0.0101 (11)	0.0048 (12)	0.0053 (10)
C14	0.0911 (12)	0.0633 (10)	0.0812 (12)	0.0077 (9)	0.0148 (10)	0.0089 (9)
C15	0.0923 (14)	0.1062 (17)	0.1091 (18)	0.0028 (13)	0.0160 (13)	0.0138 (15)
C16	0.114 (2)	0.117 (2)	0.132 (3)	−0.0128 (17)	−0.0209 (19)	0.0136 (19)
C17	0.173 (3)	0.0932 (18)	0.102 (2)	−0.002 (2)	−0.014 (2)	0.0037 (15)
C18	0.160 (3)	0.0864 (15)	0.0962 (17)	0.0159 (17)	0.0345 (17)	−0.0002 (13)
C19	0.0950 (14)	0.0792 (12)	0.1008 (16)	0.0110 (11)	0.0191 (12)	0.0042 (12)
C20	0.0897 (13)	0.0930 (14)	0.0945 (15)	0.0018 (11)	0.0043 (11)	0.0267 (12)
C21	0.0856 (12)	0.0820 (12)	0.0841 (13)	0.0078 (10)	0.0038 (10)	0.0106 (10)
C22	0.0912 (13)	0.0908 (14)	0.0825 (13)	0.0047 (11)	0.0050 (10)	0.0070 (11)
C23	0.0898 (13)	0.1191 (19)	0.0964 (15)	0.0044 (13)	0.0022 (11)	0.0204 (14)
C24	0.1070 (16)	0.148 (2)	0.0972 (17)	−0.0055 (17)	−0.0142 (13)	0.0290 (17)

### Geometric parameters (Å, º)

**Table:**

S1—C1	1.7601 (19)	C13—H13A	0.9700
S1—C6	1.771 (2)	C13—H13B	0.9700
S2—C2	1.739 (2)	C14—C19	1.377 (3)
S2—C13	1.824 (2)	C14—C15	1.385 (3)
N1—C1	1.325 (2)	C15—C16	1.381 (4)
N1—C2	1.333 (2)	C15—H15	0.9300
N2—C3	1.329 (3)	C16—C17	1.352 (5)
N2—C2	1.345 (2)	C16—H16	0.9300
N3—C5	1.140 (3)	C17—C18	1.355 (4)
C1—C4	1.396 (3)	C17—H17	0.9300
C3—C4	1.399 (3)	C18—C19	1.381 (4)
C3—C20	1.501 (3)	C18—H18	0.9300
C4—C5	1.428 (3)	C19—H19	0.9300
C6—C7	1.383 (3)	C20—C21	1.511 (3)
C6—C11	1.381 (3)	C20—H20A	0.9700
C7—C8	1.372 (3)	C20—H20B	0.9700
C7—H7	0.9300	C21—C22	1.529 (3)
C8—C9	1.384 (3)	C21—H21A	0.9700
C8—H8	0.9300	C21—H21B	0.9700
C9—C10	1.390 (3)	C22—C23	1.489 (3)
C9—C12	1.499 (3)	C22—H22A	0.9700
C10—C11	1.366 (3)	C22—H22B	0.9700
C10—H10	0.9300	C23—C24	1.518 (3)
C11—H11	0.9300	C23—H23A	0.9700
C12—H12A	0.9600	C23—H23B	0.9700
C12—H12B	0.9600	C24—H24A	0.9600
C12—H12C	0.9600	C24—H24B	0.9600
C13—C14	1.491 (3)	C24—H24C	0.9600
			
C1—S1—C6	100.16 (9)	C19—C14—C13	122.7 (2)
C2—S2—C13	102.22 (9)	C15—C14—C13	120.3 (2)
C1—N1—C2	116.55 (16)	C16—C15—C14	120.5 (3)
C3—N2—C2	116.38 (17)	C16—C15—H15	119.7
N1—C1—C4	121.56 (17)	C14—C15—H15	119.7
N1—C1—S1	118.93 (13)	C17—C16—C15	121.0 (3)
C4—C1—S1	119.50 (15)	C17—C16—H16	119.5
N1—C2—N2	126.81 (19)	C15—C16—H16	119.5
N1—C2—S2	118.82 (14)	C16—C17—C18	119.8 (3)
N2—C2—S2	114.34 (14)	C16—C17—H17	120.1
N2—C3—C4	121.19 (18)	C18—C17—H17	120.1
N2—C3—C20	117.6 (2)	C17—C18—C19	119.8 (3)
C4—C3—C20	121.2 (2)	C17—C18—H18	120.1
C1—C4—C3	117.46 (18)	C19—C18—H18	120.1
C1—C4—C5	120.89 (19)	C14—C19—C18	121.8 (3)
C3—C4—C5	121.57 (19)	C14—C19—H19	119.1
N3—C5—C4	179.3 (3)	C18—C19—H19	119.1
C7—C6—C11	119.6 (2)	C3—C20—C21	112.55 (18)
C7—C6—S1	119.71 (16)	C3—C20—H20A	109.1
C11—C6—S1	120.65 (16)	C21—C20—H20A	109.1
C8—C7—C6	119.4 (2)	C3—C20—H20B	109.1
C8—C7—H7	120.3	C21—C20—H20B	109.1
C6—C7—H7	120.3	H20A—C20—H20B	107.8
C7—C8—C9	122.0 (2)	C20—C21—C22	111.03 (18)
C7—C8—H8	119.0	C20—C21—H21A	109.4
C9—C8—H8	119.0	C22—C21—H21A	109.4
C8—C9—C10	117.3 (2)	C20—C21—H21B	109.4
C8—C9—C12	121.7 (2)	C22—C21—H21B	109.4
C10—C9—C12	121.0 (2)	H21A—C21—H21B	108.0
C11—C10—C9	121.5 (2)	C23—C22—C21	113.75 (19)
C11—C10—H10	119.3	C23—C22—H22A	108.8
C9—C10—H10	119.3	C21—C22—H22A	108.8
C10—C11—C6	120.11 (19)	C23—C22—H22B	108.8
C10—C11—H11	119.9	C21—C22—H22B	108.8
C6—C11—H11	119.9	H22A—C22—H22B	107.7
C9—C12—H12A	109.5	C22—C23—C24	112.6 (2)
C9—C12—H12B	109.5	C22—C23—H23A	109.1
H12A—C12—H12B	109.5	C24—C23—H23A	109.1
C9—C12—H12C	109.5	C22—C23—H23B	109.1
H12A—C12—H12C	109.5	C24—C23—H23B	109.1
H12B—C12—H12C	109.5	H23A—C23—H23B	107.8
C14—C13—S2	108.96 (14)	C23—C24—H24A	109.5
C14—C13—H13A	109.9	C23—C24—H24B	109.5
S2—C13—H13A	109.9	H24A—C24—H24B	109.5
C14—C13—H13B	109.9	C23—C24—H24C	109.5
S2—C13—H13B	109.9	H24A—C24—H24C	109.5
H13A—C13—H13B	108.3	H24B—C24—H24C	109.5
C19—C14—C15	117.1 (2)		
			
C2—N1—C1—C4	−0.9 (2)	C6—C7—C8—C9	0.4 (3)
C2—N1—C1—S1	178.62 (12)	C7—C8—C9—C10	1.2 (3)
C6—S1—C1—N1	−10.82 (15)	C7—C8—C9—C12	−178.7 (2)
C6—S1—C1—C4	168.75 (14)	C8—C9—C10—C11	−1.7 (3)
C1—N1—C2—N2	−0.9 (3)	C12—C9—C10—C11	178.3 (2)
C1—N1—C2—S2	−179.06 (12)	C9—C10—C11—C6	0.6 (3)
C3—N2—C2—N1	1.2 (3)	C7—C6—C11—C10	1.1 (3)
C3—N2—C2—S2	179.48 (13)	S1—C6—C11—C10	−178.29 (15)
C13—S2—C2—N1	−1.69 (16)	C2—S2—C13—C14	156.66 (16)
C13—S2—C2—N2	179.92 (14)	S2—C13—C14—C19	75.3 (2)
C2—N2—C3—C4	0.2 (3)	S2—C13—C14—C15	−104.8 (2)
C2—N2—C3—C20	−179.37 (16)	C19—C14—C15—C16	0.2 (4)
N1—C1—C4—C3	2.2 (3)	C13—C14—C15—C16	−179.7 (2)
S1—C1—C4—C3	−177.32 (13)	C14—C15—C16—C17	0.1 (5)
N1—C1—C4—C5	179.03 (17)	C15—C16—C17—C18	−0.4 (5)
S1—C1—C4—C5	−0.5 (2)	C16—C17—C18—C19	0.4 (4)
N2—C3—C4—C1	−1.9 (3)	C15—C14—C19—C18	−0.2 (3)
C20—C3—C4—C1	177.73 (17)	C13—C14—C19—C18	179.6 (2)
N2—C3—C4—C5	−178.63 (18)	C17—C18—C19—C14	0.0 (4)
C20—C3—C4—C5	1.0 (3)	N2—C3—C20—C21	−85.7 (2)
C1—S1—C6—C7	−85.01 (17)	C4—C3—C20—C21	94.7 (2)
C1—S1—C6—C11	94.37 (16)	C3—C20—C21—C22	−170.5 (2)
C11—C6—C7—C8	−1.6 (3)	C20—C21—C22—C23	−173.8 (2)
S1—C6—C7—C8	177.82 (16)	C21—C22—C23—C24	179.6 (2)
